# A topical examination of Cigar Aficionado magazine content, 2023

**DOI:** 10.18332/tpc/196229

**Published:** 2024-12-12

**Authors:** Ollie Ganz, Jennifer Cornacchione Ross

**Affiliations:** 1 Rutgers Institute for Nicotine and Tobacco Studies, Rutgers Biomedical and Health Sciences, New Brunswick, United States; 2 Department of Health Behavior, Society and Policy, Rutgers School of Public Health, Piscataway, United States; 3 Department of Health Law, Policy and Management, Boston University School of Public Health, Boston, United States

**Keywords:** tobacco control, cigars, tobacco marketing

## Abstract

**INTRODUCTION:**

Prior research has found that premium cigar marketing highlights favorable themes (e.g. glamour), and reinforces the idea that premium cigars are part of a successful, luxurious lifestyle. Yet, all but one of these studies are more than 20 years old and more recent data on premium cigar marketing is needed. This study adds to the literature by a providing a comprehensive, topical examination of all content promoted in all 2023 issues of Cigar Aficionado, a prominent cigar lifestyle magazine.

**METHODS:**

Codes were identified through a literature review and scan of issues of Cigar Aficionado from 2022. Codes included but were not limited to cigars, alcohol, travel, cigar festivals, celebrities, and cigar storage products. All coding was done in excel and then exported to Stata/MP 17 for analysis. Analysis of articles and ads were done separately.

**RESULTS:**

Among ads, the most common topics were cigars (64.5%), alcohol (31.1%), and cigar stores/retailers (14.5%). For articles, the most common topics were cigars (49.6%), cigar reviews/spotlights (23.3%), and celebrities (19.5%). Among ads for cigar products where country of origin could be identified (44.6%), most cigars featured were from Nicaragua (65.0%) and the Dominican Republic (25.2%), followed by Honduras (7.3%), Costa Rica (1.6%), and Cuba (0.8%).

**CONCLUSIONS:**

Overall, marketing in the 2023 Cigar Aficionado issues is similar to marketing strategies from the 1990s. Future research should explore health claims and other marketing strategies used in Cigar Aficionado, and other lifestyle magazines, as well as observe marketing trends over time.

## INTRODUCTION

After being outpaced by cheap, mass-merchandise cigarillos and filtered cigars for the past two decades^1,2^, premium cigars are becoming increasingly popular in the US^1,3^. Premium cigar sales hit an all-time high in 2022, with 521.4 million sticks sold^1^. Imports of premium cigars continue to increase in 2024, with about 97 million premium cigars imported in the first quarter^4^. In 2016, the Deeming Rule extended the US Food and Drug Administration’s (FDA) regulatory authority over tobacco products to include cigars^5^. Since then, political forces have sought to protect premium cigars from regulatory oversight^1,6-10^ and an August 2023 court decision stripped FDA of its authority to regulate premium cigars^11^. Given the paucity of research, the FDA commissioned the National Academies of Sciences, Engineering, and Medicine (NASEM) to evaluate the available evidence on health effects and use patterns related to premium cigars^12^. The report identified a need for more research on factors that impact premium cigar use, including marketing.

Only a handful of studies, some of which are described in the NASEM report, have examined premium cigar marketing. These studies found that the marketing highlights favorable themes (e.g. glamour and prestige), and reinforces the idea that premium cigars are part of a successful, luxurious lifestyle^12-16^. The report also concluded that while premium cigars are not heavily promoted via traditional channels (e.g. convenience store power walls) like other types of cigars, they are the focus of online marketing (e.g. brand websites) and lifestyle magazines^12^. Historically, most advertising for cigars has been in magazines, with *Cigar Aficionado* being the most prominent cigar lifestyle magazine. The 1998 National Cancer Institute’s Monograph on cigars attributes the resurgence of cigars in the early 1990s with the launching of the magazine in 1992^17^. To date, there have been few studies that have examined the magazine’s content, including both their articles and advertising, though all but one are more than 20 years old^15,16,18^. A more recent study examined the covers, every five articles, and every five advertisements for five issues of *Cigar Aficionado* published in 2021, and unsurprisingly, found that a large portion of the magazine content was cigar-specific, and that most magazine issue covers featured celebrities (including 80% male celebrities)^15^. Most cigar products advertised were domestic cigars^15^, and a smaller proportion of advertising featured alcohol (12.4%), while nearly a quarter of the ads fell into the ‘other’ category (22.8%). Given the paucity of data on premium cigar marketing and the importance of lifestyle magazines in the promotion of these products – *Cigar Aficionado* in particular – this study provides a comprehensive, topical examination of all content promoted in all 2023 issues of *Cigar Aficionado*, including identifying ‘other’ products being advertised, which could provide further insight into industry tactics to associate cigar smoking with certain lifestyles or products to make them more appealing.

## METHODS

### Magazine content

Digital issues of *Cigar Aficionado* magazine were purchased and accessed through the website, Zinio. There were six magazine issues in 2023, published bimonthly.

### Coding procedures

Themes and codes were identified based on a literature review^12-16^ and a scan of the 2022 magazine issues. Specifically, prior literature has identified the presence of alcohol, celebrities, and luxury items (e.g. fine art, expensive cars) in cigar lifestyle magazines^12,15^, as well as the promotion of cigar businesses and cigar events^16,18^. Sterling et al.^15^ also noted that both domestic and international cigar products were featured in these publications. All advertisements and articles were coded for the following topics (yes, no): cigars, alcohol, travel, sports (including car racing), cigar festivals or events, cars (not related to racing), arts (e.g. film), fashion (e.g. clothing), weapons, celebrities, cigar reviews/spotlights, premium cigar storage products and accessories (e.g. ashtray, humidor), watches/jewelry, food/recipes, consumer submitted content (e.g. letters to the editor), premium cigar stores/retailers/companies, health-related products (e.g. air filter), and policy.

We also coded for non-premium cigar festivals and product reviews, such as whiskey festivals. For ads promoting cigars, we coded for country of origin of the cigar. If more than one cigar was featured, we coded for country of origin for the first three cigars listed. Country of origin was identified by looking up the cigar on the *Cigar Aficionado* website under ‘Ratings and Reviews’^19^. Topics were not mutually exclusive. Anything pertaining to cigars, including accessories and reviews, was also coded as falling under the broad topic of cigars. Two coders each coded half of the issues. They established agreement via discussion of content of the first half of one issue, then double coded the second half of that issue to determine reliability. The coders achieved at least 90% agreement for all coded variables. Remaining issues were coded independently. Halfway through coding, they double coded another half issue to ensure continued coding agreement.

### Analysis

Issues were coded in Excel and imported into Stata/MP 17 for analysis. We used frequencies and percentages to examine the occurrences of each topic for articles and ads separately.

## RESULTS

In total, there were 276 ads and 133 articles across the six issues in 2023 (Table 1). Among ads, the most common topics were cigars (n=178; 64.5%), alcohol (n=86; 31.1%), and cigar stores/retailers (n=40; 14.5%). Other topics included non-cigar festivals (e.g. whiskey festival, n=16; 5.8%), cigar storage products (n=10; 3.5%), and weapons, such as guns or knives (n=6; 2.2%). For articles, the most common topics were cigars (n=66; 49.6%), cigar reviews/spotlights (n=31; 23.3%), and celebrities (e.g. athletes, actors, n=26; 19.5%). Other article topics included sports (n=10; 14.3%), consumer submitted content (e.g. pictures of friends smoking cigars, n=14; 10.5%), and alcohol (n=13; 9.8%).

**Table 1:** Topics covered in six issues of Cigar Aficionado, 2023

*Topics*	*Advertisements (N=276) n (%)*	*Articles (N=133) n (%)*
Cigars	178 (64.5)	66 (49.6)
Alcohol	86 (21.1)	13 (9.8)
Cigar store/retailer	40 (14.5)	2 (1.5)
Non-cigar festival	16 (5.8)	1 (0.7)
Cigar storage	10 (3.6)	6 (4.5)
Weapons	6 (2.2)	0 (0)
Cigar festival	5 (1.8)	1 (0.7)
Health-related	5 (1.8)	0 (0)
Travel	5 (1.8)	8 (6.0)
Sports	3 (1.1)	19 (14.3)
Celebrities	2 (0.7)	26 (19.5)
Food/recipes	2 (0.7)	9 (6.8)
Cars	1 (0.4)	7 (5.3)
Cigar review/spotlight	0 (0)	31 (23.3)
Art/film	0 (0)	9 (6.8)
Fashion	0 (0)	6 (4.5)
Watches/jewelry	0 (0)	4 (3.0)
Non-cigar product review	0 (0)	1 (0.7)
Policy	0 (0)	2 (1.5)
Consumer submitted content	0 (0)	14 (10.5)

Topics were not mutually exclusive.

Among ads for cigar products where country of origin could be identified (n=123; 44.6%), most cigars featured were from Nicaragua (n=80; 65.0%) and the Dominican Republic (n=31; 25.2%), followed by Honduras (n=9l; 7.3%), Costa Rica (n=2; 1.6%), and Cuba (n=1; 0.8%). One cigar in an ad was manufactured in the US. For articles, almost all cigars for which country of origin could be identified (n=27; 20.3%) were from Nicaragua (n=22; 81.5%), followed by Dominican Republic (n=3; 11.1%), Cuba (n=1; 3.7%), and Jamaica (n=1; 3.7%). Any given ad or article could feature more than one cigar, so these numbers reflect total number of cigars promoted, not total number of ads or articles.

## DISCUSSION

In our analysis of the 2023 issues of *Cigar Aficionado*, we found that most content was direct advertising (vs articles). Not surprisingly, most advertisements were related to cigars and many articles were also about cigars. Most cigars depicted were international, which contrasts with findings by Sterling et al.^15^, but aligns with recent data showing an increase in international premium cigar imports into the US^4^. This discrepancy from Sterling et al.^15^ could be due to changes in the cigars advertised from 2021 to 2023, or differences in study methods.

Other topics identified align with the Sterling et al.^15^ findings, such as alcohol (e.g. articles highlighting drink pairings with cigars), both in advertising and articles. The NASEM report concluded that alcohol use is particularly common among adults who smoke premium cigars and suggests that alcohol is part of premium cigar culture^13^. Research also shows that many festivals and events promoted in the magazine feature both premium cigars and alcohol^17^. Indeed, our study found a number of ads for whiskey festivals. The prominent promotion of alcohol in *Cigar Aficionado* raises concerns, as co-use of alcohol with tobacco is causally related to cancer risk^17^. Like Sterling et al.^15^, we also found that celebrities were prominently featured in magazine content. Celebrities have been used to sell premium cigars for decades^20^ and may be used as a tactic to appeal to non-premium cigar smokers^15^.

Our study makes several contributions to the literature. First, our analysis examined the ‘other’ category identified by Sterling et al.^15^, revealing topics outside the realm of cigars, including weapons, travel, sports, art/film, cars, food/recipes, and fashion. This range of topics may be an attempt to appeal to a broader audience beyond those who are interested in premium cigars. It may also be part of an effort to associate premium cigars with other luxury and high-end items, which aligns with how premium cigars have been promoted historically^15,17^. Further, one of the more prominent themes in our analysis of articles was consumer submitted content (e.g. letters to the editor). This type of content facilitates engagement between the magazine and its readers, leading to and reinforcing consumer loyalty, commitment, and connection to the brand^21^.

### Limitations

This study has some limitations. We coded for one years’ worth of issues, so we are not able to assess how marketing strategies may have changed or evolved over time. However, this study was comprehensive in coding recent issues and all content within each issue. Additionally, we did not examine in detail specific content or claims; rather this study provides a broad overview of the magazine’s content.

## CONCLUSIONS

Overall, marketing in the 2023 *Cigar Aficionado* issues was similar to marketing strategies from the 1990s with content for upscale products, premium alcohol, and celebrity features^13,16^ to facilitate a connection between premium cigars and luxury and glamour. Future research should examine specific health claims and other marketing strategies utilized in *Cigar Aficionado* and other lifestyle magazines, as well as monitor marketing trends over time, particularly given that premium cigars are no longer subject to FDA’s regulatory authority and marketing restrictions.
